# Structure of spin excitations in heavily electron-doped Li_0.8_Fe_0.2_ODFeSe superconductors

**DOI:** 10.1038/s41467-017-00162-x

**Published:** 2017-07-25

**Authors:** Bingying Pan, Yao Shen, Die Hu, Yu Feng, J. T. Park, A. D. Christianson, Qisi Wang, Yiqing Hao, Hongliang Wo, Zhiping Yin, T. A. Maier, Jun Zhao

**Affiliations:** 1 State Key Laboratory of Surface Physics and Department of Physics, Fudan University, Shanghai, 200433 China; 2 Heinz Maier-Leibnitz Zentrum (MLZ), Technische Universität München, Garching, D-85748 Germany; 3 Quantum Condensed Matter Division, Oak Ridge National Laboratory, Oak Ridge, Tennessee 37831-6393 USA; 4 Department of Physics and Astronomy, University of Tennessee, Knoxville, Tennessee 37996 USA; 5 Department of Physics and Center for Advanced Quantum Studies, Beijing Normal University, Beijing, 100875 China; 6 Computer Science and Mathematics Division and Center for Nanophase Materials Sciences, Oak Ridge National Laboratory, Oak Ridge, Tennessee 37831 USA; 7 Materials Science and Technology Division, Oak Ridge National Laboratory, Oak Ridge, Tennessee 37831 USA; 8 Collaborative Innovation Center of Advanced Microstructures, Nanjing, 210093 China

## Abstract

Heavily electron-doped iron-selenide high-transition-temperature (high-*T*_c_) superconductors, which have no hole Fermi pockets, but have a notably high *T*_c_, have challenged the prevailing *s*_±_ pairing scenario originally proposed for iron pnictides containing both electron and hole pockets. The microscopic mechanism underlying the enhanced superconductivity in heavily electron-doped iron-selenide remains unclear. Here, we used neutron scattering to study the spin excitations of the heavily electron-doped iron-selenide material Li_0.8_Fe_0.2_ODFeSe (*T*_c_ = 41 K). Our data revealed nearly ring-shaped magnetic resonant excitations surrounding (*π*, *π*) at ∼21 meV. As the energy increased, the spin excitations assumed a diamond shape, and they dispersed outward until the energy reached ∼60 meV and then inward at higher energies. The observed energy-dependent momentum structure and twisted dispersion of spin excitations near (*π*, *π*) are analogous to those of hole-doped cuprates in several aspects, thus implying that such spin excitations are essential for the remarkably high *T*_c_ in these materials.

## Abstract (web summary)

The microscopic mechanism underlying an enhanced superconductivity in electron-doped iron selenide superconductor remains unclear. Here, Pan et al. report the spin excitations of Li_0.8_Fe_0.2_ODFeSe, revealing analogous momentum structure and dispersion to hole-doped cuprates.

## Introduction

Quantitative knowledge of the energy and momentum dependence of the spin excitations of high-temperature superconductors is essential in establishing the mechanism underlying superconductivity. In iron-pnictide superconductors, it is widely believed that the electron pairing is mediated by the stripe spin fluctuations near the nesting wavevector (*π*, 0) between the hole pockets at the Brillouin zone center and the electron pockets at the zone edges (1-Fe unit cell), which typically leads to an *s*-wave pairing with a sign-reversed gap function (*s*_±_)^1^. However, in the parent phase of iron senlenide superconductors FeSe (*T*_c_ = 8.7 K), in addition to the stripe spin fluctuations near (*π*, 0), Néel spin fluctuations near (*π*, *π*) were observed over a wide energy range^2^. Moreover, the *s*_±_ pairing scenario is directly challenged by heavily electron-doped iron-selenides (HEDIS) because these superconductors contain only electron pockets, but not hole pockets^3–10^. In particular, the wavevector connecting the electron pockets is close to (*π*, *π*) rather than (*π*, 0) in HEDIS, and this raises noteworthy questions as to how the spin excitation and pairing symmetry evolve as the system is tuned into the heavily electron-doped regime in which superconductivity is surprisingly enhanced^6, 11–13^.

The pairing symmetry in HEDIS is currently under intense theoretical debate^14–25^. In experimental studies, scanning tunneling microscopy quasiparticle interference measurements suggested a sign-preserved *s*-wave superconducting gap function between electron pockets in single-layer FeSe/SrTiO_3_ thin films^26^, whereas low-energy inelastic neutron scattering data revealed a magnetic resonant mode around (*π*, 0.5*π*) in A_*x*_Fe_2−*y*_Se_2_ (A = K, Rb…), indicating a sign-reversed superconducting gap function^27–29^. However, the lack of phase-pure single-crystalline samples have rendered impractical the measurement of the momentum structure of spin excitations over a wider energy range throughout the entire Brillouin zone in any HEDIS superconductor. In addition, studies have argued that the coexisting $\sqrt 5$ × $\sqrt 5$ iron vacancy ordered antiferromagnetic insulating phase in A_*x*_Fe_2−*y*_Se_2_ and the substrate of the single-layer FeSe/SrTiO_3_ may influence superconductivity^30, 31^. As yet a complete picture of the spin excitations and the pairing symmetry of a pure bulk single-crystalline HEDIS material remain unclear.

The newly discovered HEDIS Li_0.8_Fe_0.2_OHFeSe (*T*_c_ = 41 K) exhibits remarkably similar electronic and superconducting gap structures to those of single layer FeSe/SrTiO_3_^9, 10, 32, 33^. Particularly, because the phase-pure single-crystalline Li_0.8_Fe_0.2_OHFeSe with a sufficiently large size can be grown, the corresponding intrinsic bulk properties not subject to the influence of the interface or impurity phases can be investigated.

In this paper, we report neutron scattering measurements of the spin excitations over a wide range of momentum and energy in single-crystalline Li_0.8_Fe_0.2_ODFeSe (*T*_c_ = 41 K) (“Methods”). Our data revealed nearly ring-shaped magnetic resonant excitations at ~21 meV, comprising four elliptical peaks at (*π*, 0.62*π*) and equivalent wavevectors surrounding (*π*, *π*). The presence of the resonance mode surrounding (*π*, *π*) indicates a sign reversal in the superconducting gap function between the electron pockets. As the energy increased, the spin excitations assumed a diamond shape, and they dispersed outward until the energy reached ~60 meV and then inward at higher energies, eventually forming a big blob near (*π*, *π*) at 130 meV. Such an energy-dependent momentum structure and twisted dispersion of spin excitations near (*π*, *π*) resemble to those in hole-doped cuprates in several aspects^34–37^. Our results imply that such spin excitations are an essential ingredient for high temperature superconductivity in these materials.

## Results

### Magnetic resonance mode

**Fig. 1:**
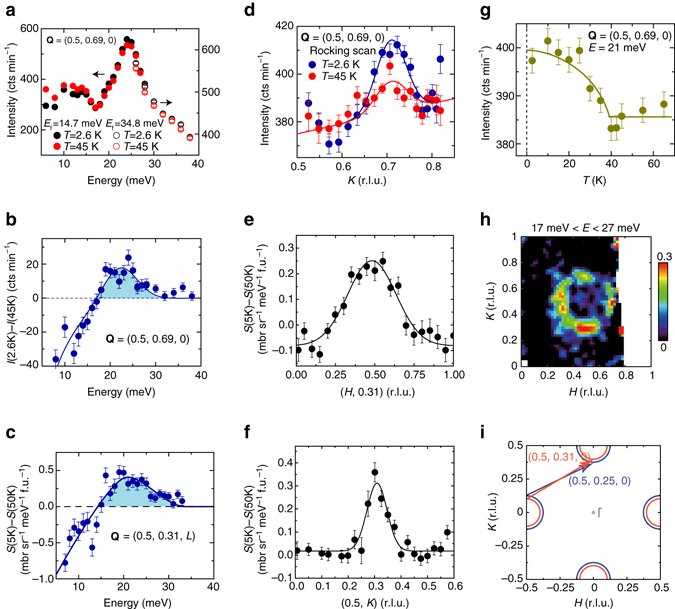
Magnetic resonant mode in Li_0.8_Fe_0.2_ODFeSe (*T*_c_ = 41 K). **a** Energy dependence of spin excitations for Li_0.8_Fe_0.2_ODFeSe at **Q** = (0.50, 0.69, 0) in the superconducting state (*T* = 2.6 K) and normal state (45 K). The *solid* and *open circles* correspond to the data collected at final energies of *E*_f_ = 14.7 and 34.8 meV, respectively. **b** Intensity difference between the superconducting state and normal state (*S* (2.6 K)–*S* (45 K)) at (0.50, 0.69, 0) measured on the PUMA thermal triple-axis spectrometer. The data collected at different *E*_f_ were normalized. **c** Intensity difference between the superconducting state and normal state (*S* (5 K)–*S* (50 K)) at (0.50, 0.31, *L*) measured on the ARCS time of flight spectrometer (0.5 ≤ *L* ≤ 4). **d** Rocking scan near (0.50, 0.69, 0) at *E* = 21 meV at T = 2.6 K and 45 K. **e**, **f** Intensity difference between the superconducting state and normal state (*S* (5 K)–*S* (50 K)) along the (*H*, 0.31) and (0.50, *K*) directions. **g** Temperature dependence of the scattering at (0.50, 0.69, 0) and *E* = 21 meV. **h** Intensity difference image (*S* (5 K)–*S* (50 K)) at *E* = 22 ± 5 meV. The *color bar* indicates scattering intensity in unit of mbr sr^−1^ meV^−1^ f. u.^−1^. The *white regions* in the *color plot* are gaps between neutron detectors. **i** Schematic of the electron Fermi pockets in Li_0.8_Fe_0.2_ODFeSe (*red*) and (Tl,Rb)_*x*_Fe_2−*y*_Se_2_ (*blue*) adapted from ref. ^9^. The calculated resonance wavevector is approximately (0.50, 0.3125) for 0.1 electrons per Fe^14^, which is consistent with our data (0.50, 0.31). The *error bars* indicate one standard deviation

We first used the PUMA thermal triple-axis spectrometer to measure low-energy spin excitations and their interplay with superconductivity. Figure 1a illustrates the energy scans at **Q** = (0.5, 0.69, 0) in the superconducting state (*T* = 2.6 K) and normal state (*T* = 45 K), indicating that the scattering at ~21 meV is enhanced below *T*_c_, whereas that at lower energies is suppressed below *T*_c_. To ensure a clear illustration of the effect of superconductivity, Fig. 1b presents the plot of the intensity difference associated with the energy scans between the superconducting state and normal state, revealing a spin-gap like feature below 18 meV and a resonance mode at ~21 meV below the superconducting gap (2Δ ≈ 28 meV)^9, 38^. A similar resonance mode at ~20 meV is also revealed near **Q** = (0.5, 0.31, *L*) in the data measured on the ARCS time-of-flight (TOF) chopper spectrometer (Fig. 1c). This is consistent with a spin exciton within the superconducting gap when the gap function exhibited an opposite sign between the electron pockets at the adjacent zone edges^14^.

The resonance excitation at 21 meV is further confirmed by the **Q-**scans near (0.5, 0.69, 0) across *T*_c_. Figure 1d shows that the normal-state spin excitation (*T* = 45 K) can be described by a single Gaussian peak on a linear background and that it is significantly enhanced on entering the superconducting state (*T* = 2.6 K). Figure 1g illustrates the detailed temperature dependence of the scattering, again confirming that the enhancement of the spin excitations is tightly associated with superconductivity and behaves like an order parameter below *T*_c_.

To precisely determine the momentum structure of the resonance mode, we used the ARCS spectrometer to map out the Brillouin zone (“Methods”). The intensity difference image between the superconducting state and normal state [*S* (5 K)−*S* (50 K)] near the resonance energy (Fig. 1h) showed elliptical peaks at (0.5, 0.5 ± *δ*) and (0.5 ± *δ*, 0.5) symmetrically surrounding (0.5, 0.5), forming a nearly ring-like structure. The peak position (0.5, 0.5 ± 0.19) and the anisotropy of the scattering in momentum space were further confirmed by constant-energy cuts, revealing a significant difference between the peak widths along the *H* and *K* directions (Fig. 1e, f). We noted that the observed incommensurability *δ* = 0.19 was smaller than that observed (*δ* ≈ 0.25) for the resonance mode in A_*x*_Fe_2−*y*_Se_2_^27–29^. This is probably because the electron doping level in Li_0.8_Fe_0.2_ODFeSe (~0.08–0.1 electrons per Fe) is lower (less overdoped) than that in A_*x*_Fe_2−*y*_Se_2_ (~0.18 electrons per Fe)^9^. Therefore, the low-energy magnetic scattering in Li_0.8_Fe_0.2_ODFeSe is closer to (*π*, *π*) compared with that in A_*x*_Fe_2−*y*_Se_2_ (Fig. 1h, i).

### Momentum and energy dependence of high-energy spin excitations

**Fig. 2:**
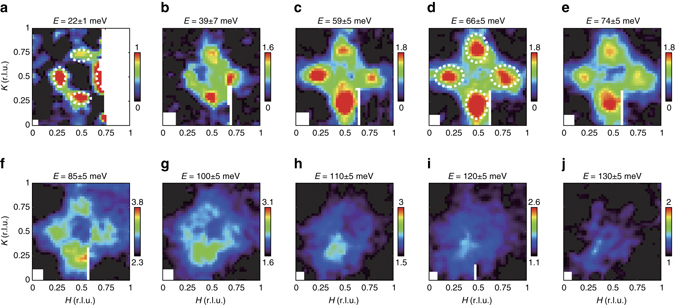
Momentum dependence of the spin excitations in Li_0.8_Fe_0.2_ODFeSe at *T* = 5 K. **a**–**j** Constant-energy images acquired at 5 K and at indicated energies. |**Q**|-dependent background was subtracted in **a**–**e** (Supplementary Note 4). For energies ≥85 meV, raw data are presented, **f**–**j**. The measurements in **a** and **b**–**j** were conducted at the incident neutron energies of 49.6 and 191.6 meV, respectively. Symmetry equivalent data were collected and averaged to enhance statistical accuracy. The *color bar* indicates scattering intensity in unit of mbr sr^−1^ meV^−1^ f. u.^−1^

To acquire a complete picture of the spin excitations, we present in Fig. 2 the evolution of the spin response with energy. At low energies, the momentum structure of the spin excitations was similar to that of the resonance mode (Fig. 2a); as the energy increased, the magnetic response assumed a diamond shape and dispersed outward (Figs. 2b–d). Concurrently, the major axis of the elliptical peaks was rotated by 90° at energies of ~59–66 meV with respect to the axis of those observed at lower energy (marked by *dashed ellipses* in Fig. 2a, d; the rotation of the elliptical peaks is further illustrated in Supplementary Fig. 1). When the energy exceeded 66 meV, the scattering dispersed inward and formed a nearly ring-like pattern at 100 meV, and eventually becoming a big blob near (0.5, 0.5) at 130 meV (Fig. 2e–j). We note that a weak ferromagnetic peak near *Q* ≈ 0, which likely arises from the Li–Fe layer, was revealed by small angle neutron scattering measurements in polycrystalline (^7^Li_0.82_Fe_0.18_OD)FeSe (*T*_c_ = 18 K)^39^. This wavevector (*Q* ≈ 0) was not covered in our measurements in the first Brillouin zone. We also did not observe clear ferromagnetic excitations in the second Brillouin zone, which could be due to the intrinsic weak signal and the decreased magnetic form factor. It is also possible that our sample which has a different chemical composition and a higher *T*_c_ (41 K) has weaker or no ferromagnetic correlations.

**Fig. 3:**
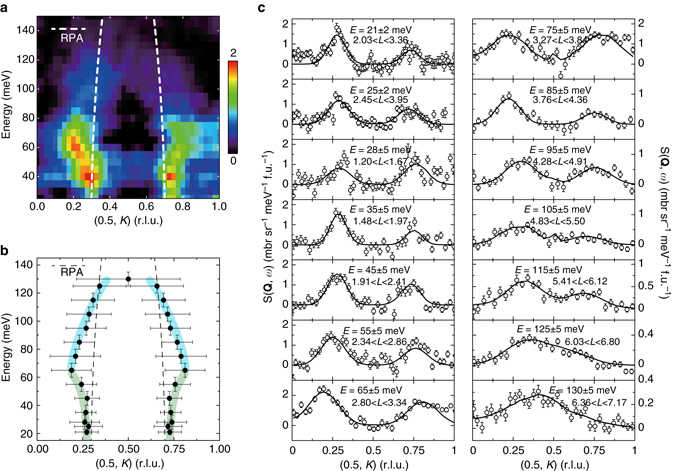
Dispersion of the spin excitations in Li_0.8_Fe_0.2_ODFeSe at *T* = 5 K. **a** Background subtracted *E*-*K* slice with incident neutron energy of 191.6 meV. A twisted dispersion is clearly illustrated. The incident neutron beam was parallel to the *c* axis and *L* was coupled with the energy transfer. No *L* modulations were observed from the scattering, indicating a two-dimensional nature of the magnetism. The *color bar* indicates scattering intensity in unit of mbr sr^−1^ meV^−1^ f. u.^−1^. **b** Dispersion acquired from the constant energy cuts in **c**. The *horizontal error bars* represent the full-width at half-maximum of the Gaussian peaks in **c**; the *vertical error bars* represent the energy integration interval. The curves are guides to the eye. **c** Constant energy cuts along the (0.5, *K*) direction at the indicated energies and energy/*L* integration interval. The measurements were conducted at incident neutron energies of 49.6 and 191.6 meV. The peak positions are determined by fitting with Gaussian profiles convoluted with the instrumental resolution, with a correction of the Fe^2+^ magnetic form factor. The *error bars* indicate one standard deviation. We note that the magnetic intensities in **a**, **c** and Fig. 2 are asymmetric because the magnetic form factor drops at larger |**Q**|

The overall dispersion of the spin excitations in Li_0.8_Fe_0.2_ODFeSe can be seen more clearly in *E*-**Q** space. As the energy increased, the magnetic excitations dispersed outward and then inward (Fig. 3a, b). This dispersion is further confirmed by the constant-energy cuts along the *K* direction at various energies in Fig. 3c. Such a strongly energy-dependent momentum structure and twisted dispersions of spin excitations are in analogy to those of hole-doped cuprate superconductors, which typically demonstrate the existence of inflection/saddle points in the dispersion curves and the rotation of the scattering pattern across inflection/saddle points^34–36^. On the other hand, spin excitations in most iron-pnictide superconductors generally disperse outward from (*π*, 0) to (*π*, ± *π*) which can usually be described by either an itinerant or local moment model^1^, or the density functional theory (DFT) combined with dynamical mean field theory (DMFT) taking into account both correlation effects and realistic band structures^40^. It should be noted that FeTe-based materials also display unusual hourglass-like dispersions^41–43^. However, the magnetic interaction in FeTe-based materials is very complicated because of the spiral magnetism induced by the interstitial Fe^41^ and the competition between the double stripe (*π*/2, *π*/2) and stripe (*π*, 0) magnetism^40, 44^.

### Momentum integrated local susceptibility

**Fig. 4:**
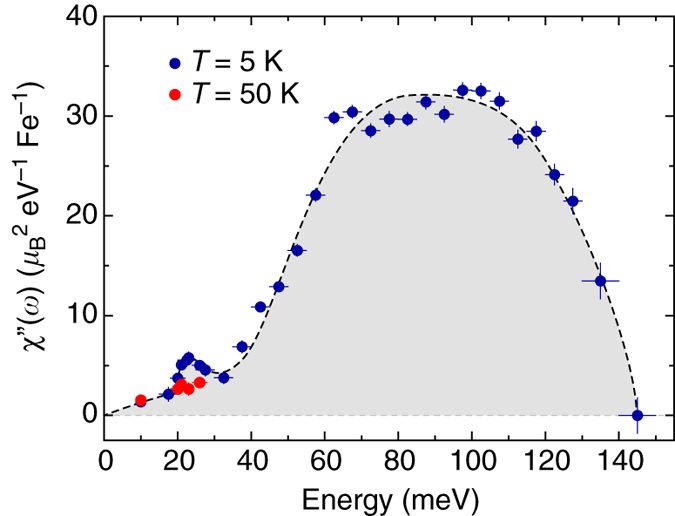
Energy dependence of the dynamic local susceptibility of Li_0.8_Fe_0.2_ODFeSe at *T* = 5 and 50 K. The *vertical* and *horizontal bars* represent the standard deviation and integrated energy interval, respectively. The *dashed curves* are guides to the eye

The use of phase-pure single crystals enables executing quantitative spin-excitation measurements in absolute units, which has not been feasible for phase-separated A_*x*_Fe_2−*y*_Se_2_. Figure 4 shows the energy dependence of the local dynamic susceptibility of Li_0.8_Fe_0.2_ODFeSe in absolute units, revealing a two-peak structure, which is similar to those of iron pnictides and FeSe^1, 2, 45, 46^, although the momentum structures of the spin excitations in these materials are quite different. The peak of the lower energy component corresponds to the resonance mode; the higher energy component, which carries much more spectral weight, peaked near 60–110 meV (Fig. 4). Notably, the integrated resonance spectral weight ($\chi _{5{\rm{K}}}\!\!\!\!\!\prime \!\prime \,\,- \chi _{50{\rm{K}}}\!\!\!\!\!\!\!\prime \!\prime$     = 0.029(7) $\mu _{\rm{B}}^2$ Fe^−1^) in Li_0.8_Fe_0.2_ODFeSe is at least one order of magnitude larger than that in bulk FeSe (*T*_c_ = 8.7 K). However, the high energy component of the spectral weight is much lower than that in FeSe^2, 47^. The effect of the electron-doping seems to entail the considerable suppression of high-energy responses and enhancement of low-energy responses. This behavior resembles that of hole-doped cuprates and hole-doped iron pnictides^36, 45^, but differs from that of the electron-doped iron pnictides in which the high-energy spin excitations were essentially doping independent and the superconductivity was completely suppressed in the over electron-doped regime^45^. The spin excitation band widths of Li_0.8_Fe_0.2_ODFeSe and FeSe are lower than those of most iron pnictides, indicating stronger electron correlations in FeSe-based superconductors^40^.

### BCS/RPA calculations and DFT + DMFT calculations

**Fig. 5:**
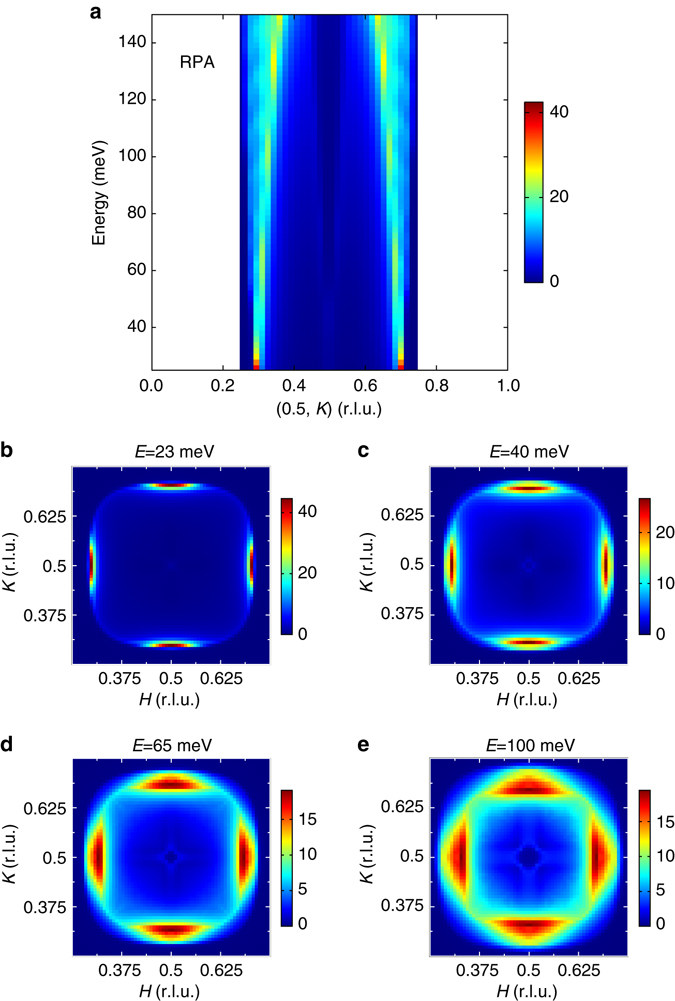
Calculated dispersion of the spin excitations of Li_0.8_Fe_0.2_ODFeSe in the superconducting state. The calculation was done using a BCS/RPA approach described in the main text. **a** Dispersion of the spin excitations. **b**–**e** Momentum structure of the spin excitations at indicated energies. The *color bars* indicate intensity in arbitrary unit

The magnetism in iron-based materials may arise from either the exchange interaction of the local moments or Fermi surface nesting of itinerant electrons, or both^1^. To the best of our knowledge, the observed energy-dependent momentum structure and twisted dispersion of the spin excitations have not been predicted in the known theoretical calculations in HEDIS based on either a local or itinerant picture. Using a BCS/RPA approach^14^, we have calculated the magnetic susceptibility from a 2D tight-binding five-orbital Hubbard–Hund Hamiltonian that describes the electronic structure of an FeSe system with only electron pockets^14^. For the superconducting gap, we have used a phenomenological ${d_{{x^2} - {y^2}}}$ gap $\Delta (k) = {\Delta _0}\left( {{\rm{cos}}\,{k_x} - {\rm{cos}}\,{k_y}} \right)$, which is close to isotropic on the electron pockets and changes sign between them. Previous calculations have shown that this type of gap structure leads to a neutron resonance in *χ*(*q*, *ω*) near *q* ≈ (*π*, 0.6*π*) that arises from scattering between the electron pockets on which the gap changes sign^14, 48^. The interaction matrix in orbital space used in the RPA calculation contains on-site matrix elements for the intra-orbital and inter-orbital Coulomb repulsions *U* and *U*′, and for the Hunds-rule coupling and pair-hopping terms *J* and *J*′, respectively. Here, we have used spin-rotationally invariant parameters *J* = *J*′ = *U*/4 and *U*′ = *U*/2 with *U* = 0.96 eV. Figure 5a shows the calculated dispersion of spin excitations as the energy increases and Fig. 5b–e displays its momentum structure for different energies. As one sees, the momentum position of the magnetic excitations below 40 meV is broadly consistent with the experiments (Figs. 3a, b and 5a). However, the BCS/RPA calculations fail to describe the outward dispersion at higher energies seen in Fig. 3a, b as well as the twisted momentum structure seen in Fig. 2. We also used a combination of DFT and DMFT, so called DFT + DMFT as implemented in ref. ^40^ to compute the electronic structure and spin dynamics of this compound (Supplementary Fig. 2; Supplementary Note 1). Similar to the BCS/RPA calculations, the DFT + DMFT-calculated spin excitation spectra also only show inward dispersion, and no twisted structure is observed (Supplementary Fig. 2i).

## Discussion

The distinct dispersion of spin excitations below and above ~60 meV might imply different origins of these excitations. The low-energy spin excitations are possibly related to Fermi surface nesting, as the resonance wavevector and its doping dependence agree with our BCS/RPA calculations^9, 14, 27^; whereas, the high-energy spin excitations, which carry more spectral weight, are possibly due to vestigial short-range magnetic exchange interactions, as also observed in hole-doped cuprates^36, 37^. Regardless of their origins, the spin excitations surrounding (*π*, *π*) as well as the electron Fermi pockets connected by (*π*, *π*) may interact collaboratively to enhance superconductivity. These structures of spin excitations and Fermi surfaces are analogous to those of hole-doped cuprates, thus implying that such spin excitations are a key ingredient for the remarkably high *T*_c_ in these materials.

Recently, we noticed a related paper describing low-energy spin excitations on Li_1−*x*_Fe_*x*_ODFe_1−*y*_Se powder samples^49^.

## Methods

### Sample growth and characterizations

Li_0.8_Fe_0.2_ODFeSe single crystals were grown using a hydrothermal method similar to that described in ref. ^50^, and were deuterated to reduce the incoherent scattering from hydrogen atoms for the inelastic neutron scattering measurements. Magnetic susceptibility and resistivity measurements conducted on a crystal from the same batch as those measured with neutrons showed a sharp superconducting transition at 41 K, signifying the high quality of the crystal. The single crystalline sample was also ground into powder for the X-ray diffraction refinements (Supplementary Fig. 3; Supplementary Note 2; Supplementary Table 1). The refined structure parameters are consistent with those in previous reports^32^.

### Inelastic neutron scattering experiments

Inelastic neutron scattering measurements were carried out on the PUMA thermal neutron triple-axis spectrometer (TAS) at the Heinz Maier–Leibnitz Zentrum (MLZ), TU München, Germany, and the ARCS TOF chopper spectrometer at the Spallation Neutron Source of Oak Ridge National Laboratory, USA (Supplementary Note 3). For the TAS experiment, ~30 pieces of single crystals with a total mass of 3.2 g were coaligned in the (*H K* 0) plane to a mosaicity within ~4°. For the TOF experiment, 8.5 g crystals were coaligned to a masaicity within ~5°; the incident beam was parallel to the *c*-axis. The wavevector **Q** at (*q*_*x*_, *q*_*y*_, *q*_*z*_) is defined as (*H*, *K*, *L*) = (*q*_*x*_ *a*/2*π*, *q*_*y*_ *a*/2*π*, *q*_*z*_ *c*/2*π*) reciprocal lattice units (r.l.u.) in the 1-Fe unit cell. Here, (0.5, 0.5) and (0.5, 0) correspond to (*π*, *π*) and (*π*, 0), respectively. The |**Q**|-dependent background was subtracted for the data in Fig. 2a–e following the practice of ref. ^2^ (Supplementary Fig. 4; Supplementary Note 4). Additional low-energy data are shown in Supplementary Fig. 5 (Supplementary Note 5). The scattering intensity was normalized into absolute units by calibrating it against the incoherent scattering of a standard vanadium sample.

### Data availability

All data that support the findings of this study are available from the corresponding authors upon request.

## Electronic supplementary material

Supplementary Information
