# Supplementary material for: Structure of spin excitations in heavily electron-doped Li0.8Fe0.2ODFeSe superconductors
Source: Nat Commun. 2017 Jul 25;8:123. doi: 10.1038/s41467-017-00162-x (PMC5527112; doi:10.1038/s41467-017-00162-x)
Supplement: Supplementary file 1 — Supplementary Information [file 41467_2017_162_MOESM1_ESM.pdf]

File name: Supplementary Information
Description: Supplementary Figures, Supplementary Table, Supplementary Notes and Supplementary References

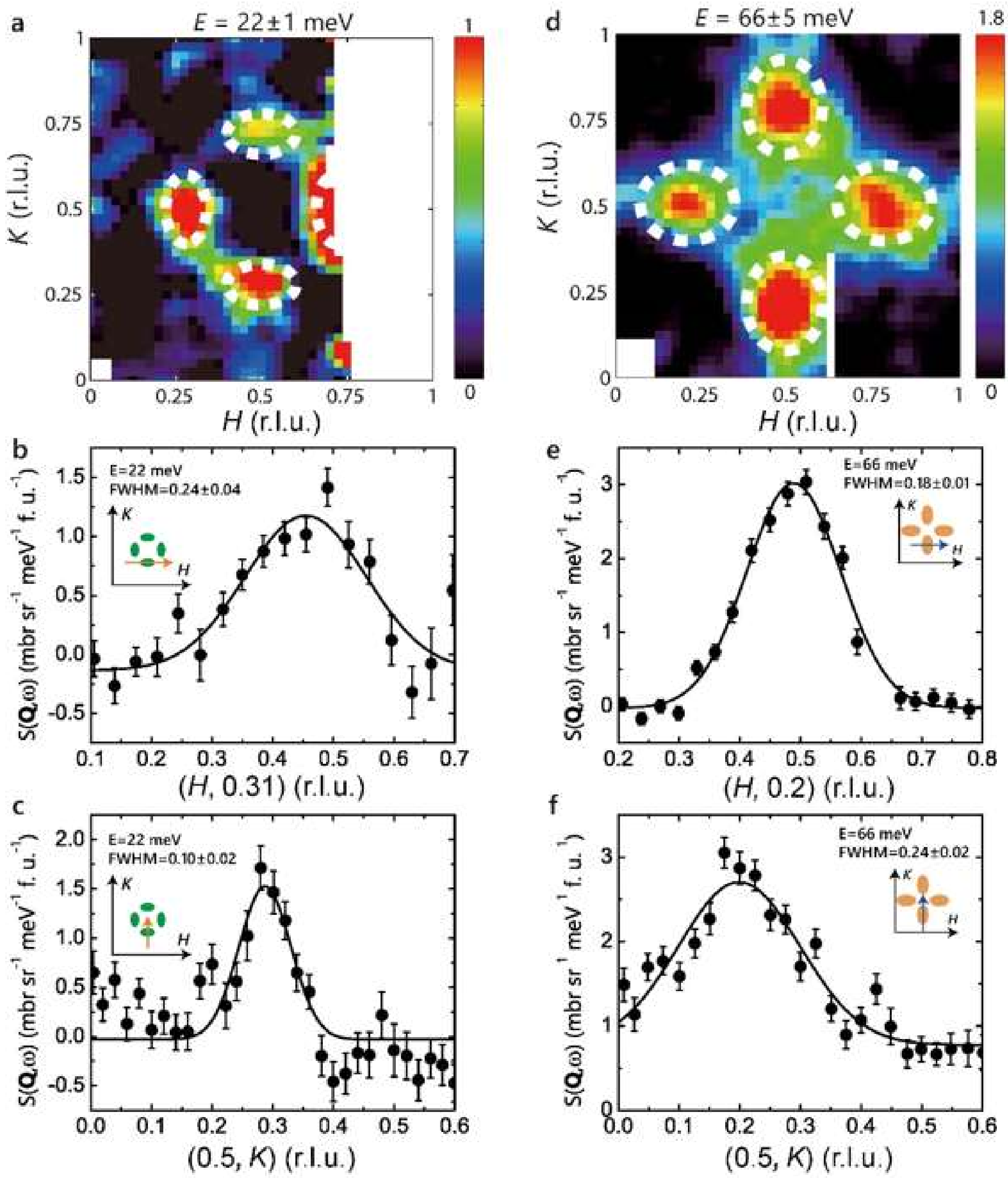

Supplementary Figure 1: Rotation of the spin excitation pattern. (a) Constant energy image at 22 meV. **(b, c)** Constant-energy cuts along the *H* (**b**) and *K* (**c**) directions at 22 meV. **(d)** Constant energy image at 66 meV. **(e**, **f)** Constant-energy cuts along the *H* (**e**) and *K* (**f**) directions at 66 meV. The insets in (**b,c,e,f**) indicate the cutting directions. The constant-energy cuts at 22 meV in **(b,c)** show that the full-width-at-half-maximum (FWHM) of *H* cut (0.24$\pm$0.04 r.l.u.) is larger than that (0.10$\pm$0.02 r.l.u.) of *K* cut. Here, r.l.u. represents reciprocal lattice units. On the other hand, at 66 meV the FWHM of *H* cut (0.18$\pm$0.01 r.l.u.) is smaller than that (0.24$\pm$0.02 r.l.u.) of *K* cut, as shown in **(e,f)**. This clearly shows that the major axis of the elliptical peaks at 66 meV was rotated by 90° with respect to the axis of those at 22 meV. The color bars indicate absolute scattering intensity in unit of mbr sr^{-1} meV^{-1} f. u.$^{-1}$. The error bars indicate one standard deviation.

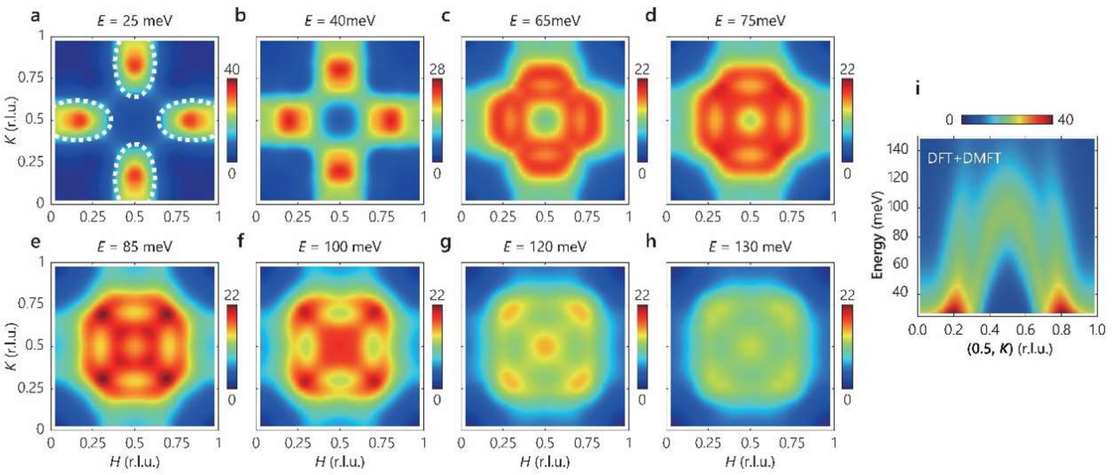

Supplementary Figure 2: DFT+DMFT calculations of the spin excitation spectra in $Li_{0.8}Fe_{0.2}ODFeSe$. a-h, Momentum structure of the spin excitations at the indicated energies. **i**, Dispersion of the spin excitations. The calculations show four longitudinal elongated elliptical peaks at 25 meV, marked by dashed ellipses in **(a)**. The calculated dispersions are shown in **(i)** which only show inward dispersion, and no twisted structure is observed. The color bars indicate intensity in arbitrary unit.

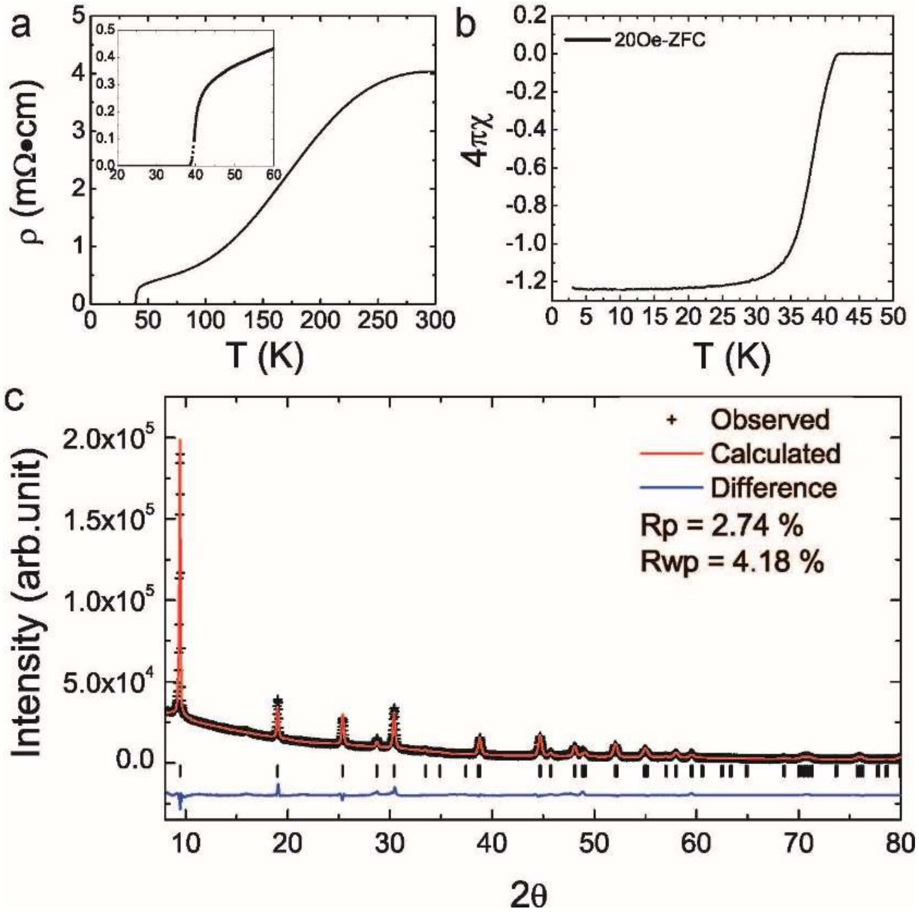

Supplementary Figure 3: Characterization of $Li_{0.8}Fe_{0.2}ODFeSe$ single crystals. (a) In-plane resistivity of the $Li_{0.8}Fe_{0.2}ODFeSe$ single crystal. **(b)** Zero-field-cooling (ZFC) magnetic susceptibility under magnetic field of 20 Oe applied perpendicular to the *c* axis. The magnetic shielding fraction is close to 100%, indicating bulk superconductivity. **(c)** Powder X-ray diffraction pattern and refinements of ground $Li_{0.8}Fe_{0.2}ODFeSe$ single crystals.

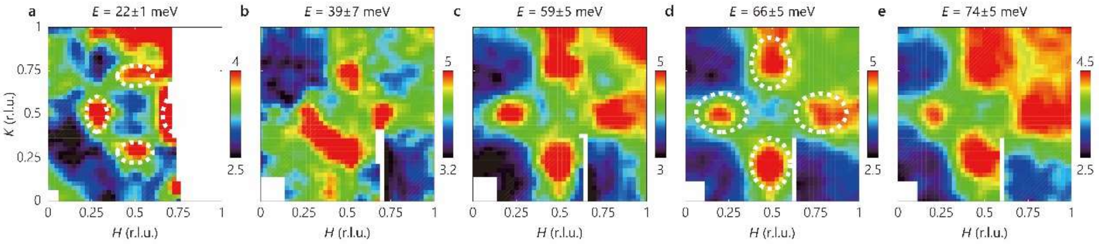

Supplementary Figure 4: Raw constant-energy images at the indicated energies. The selected energies are from **(a)** 22±1 meV to **(e)** 74±5 meV. The white ellipses in **(a)** and **(d)** clearly show the rotation of spin excitation pattern. The color bars indicate absolute scattering intensity in unit of mbr sr^{-1} meV^{-1} f. $u.^{-1}$.

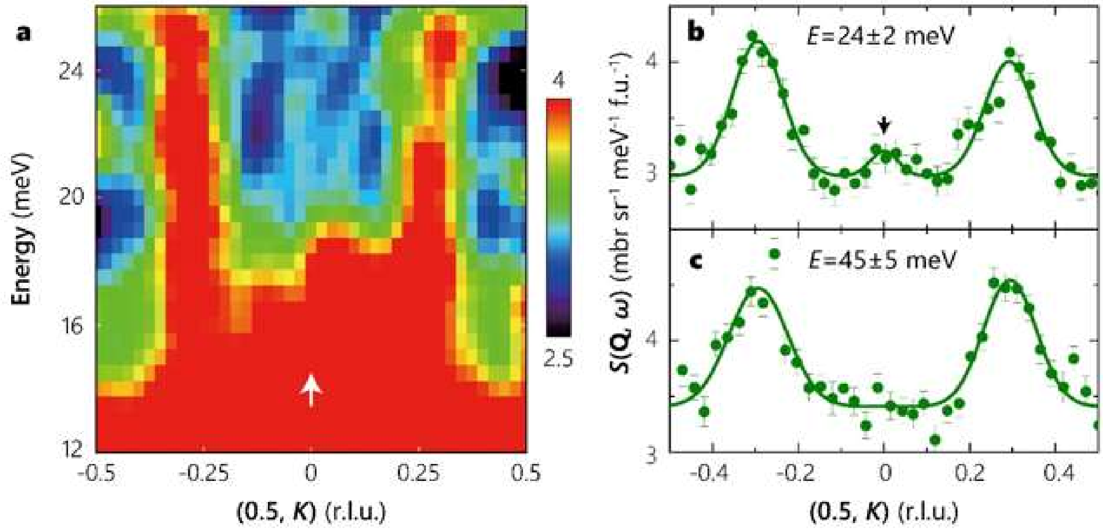

Supplementary Figure 5: Low energy excitation spectrum at 5 K. (a) *E*-*K* slice with incident neutron energy of E_{i} = 49.6 meV at 5 K. The white arrow indicates the weak excitations near (0.5, 0). The color bars indicate absolute scattering intensity in unit of mbr sr^{-1} meV^{-1} f. $u.^{-1}$. **(b, c)** Constant-energy cuts along the *K* direction at *E* = 24 meV (**b**) and 45 meV (**c**). The excitations at (0.5, 0) are no longer discernable at 45 meV. The measurements in (**b**) and (**c**) were conducted at incident energies of 49.6 and 191.6 meV, respectively. The error bars indicate one standard deviation.

Supplementary Table 1. Refined structural parameters for $Li_{0.8}Fe_{0.2}ODFeSe$ at room temperature.

Space group	*P*4/*nmm*				
a (Å)	3.78156 (9)				
c (Å)	9.31139(43)				
χ^2	16.2				
Rp	2.74				
Rwp	4.18				
Atom	*x*	*y*	*z*	B_{iso}	*occupancy*
Li	0.75000	0.25000	0.00000	1.094	0.101
Fe1	0.75000	0.25000	0.00000	1.094	0.024
O	0.25000	0.25000	-0.05709(141)	1.094	0.125
D	0.25000	0.25000	0.17400	1.094	0.125
Fe2	0.75000	0.25000	0.50000	0.705(143)	0.125
Se	0.25000	0.25000	0.34190(38)	0.273(112)	0.125

Supplementary Note 1: DFT+DMFT calculations for the spin excitations in $Li_{0.8}Fe_{0.2}ODFeSe$

We use a combination of density functional theory (DFT) and dynamical mean field theory (DMFT), so called DFT+DMFT[1] as implemented in ref. 2 to compute the electronic structure and spin dynamics of this compound. The DFT part is based on the full-potential linear augmented plane wave method implemented in Wien2K[3] in conjunction with a generalized gradient approximation[4] of the exchange correlation functional. In the DFT+DMFT calculations, the electronic charge was computed self-consistently on DFT+DMFT density matrix. The quantum impurity problem was solved by the continuous time quantum Monte Carlo (CTQMC) method[5, 6], using Slater form of the Coulomb repulsion in its fully rotational invariant form. In consistent with previous publication[7,8], We use a Hubbard U=5.0 eV and Hund's rule coupling J=0.8 eV, and experimentally determined crystal structure including the internal positions of the atoms as shown in Supplementary Table 1. The bare susceptibility was computed using the fully self-consistent DFT+DMFT lattice Green's function and the spin susceptibility was computed using the Bethe-Salpeter equation that takes into account two-particle vertex correction. Here the two-particle (particle-hole) irreducible vertex is local within DMFT and it is equal to the impurity vertex, which can be obtained from the solution of the quantum impurity model using CTQMC. Further computational details on spin susceptibility are available in ref. 8.

Supplementary Note 2: Characterization of $Li_{0.8}Fe_{0.2}ODFeSe$ single crystals

$Li_{0.8}Fe_{0.2}ODFeSe$ single crystals were grown using a hydrothermal method similar to that described in ref. 9. Magnetic susceptibility and resistivity measurements conducted on a crystal from the same batch as those measured with neutrons showed a sharp superconducting transition at 41 K (Supplementary Fig. 3a, b). Powder X-ray diffraction refinements on ground single crystals confirmed a pure tetragonal phase with no detectable impurities. The refined structure parameters are summarized in Supplementary Table 1, which are consistent with previous reports[10].

Supplementary Note 3: Inelastic neutron scattering experiments

Inelastic neutron scattering experiments were carried out on the PUMA thermal neutron triple-axis spectrometer at Heinz Maier-Lerbnitz Zentrum (MLZ), TU München, Germany, and the ARCS time-of-flight chopper spectrometer at the Spallation Neutron Source of Oak Ridge National Laboratory, USA. For the measurements on PUMA (Fig. 1a, 1d, 1g and 1b), we used the pyrolytic graphite

[PG(002)] as the monochromator and analyzer. The monochromator was double focused. A PG filter was installed in front of the analyzer to eliminate higher order neutrons. For the measurements on ARCS (Fig. 1c, 1e, 1f, 1h, Fig. 2-4), incident energies of E_i = 191.6 and 49.6 meV were used. The color plots of the TOF data were visualized using the MSLICE program[11] in which data were smoothed by repeated normalized convolution with a 3x3 matrix [0.1 0.2 0.1; 0.2 0.8 0.2; 0.1 0.2 0.1].

Supplementary Note 4: Background subtraction for TOF data

Strong phonon background can be seen at high |**Q**| in the raw constant-energy images at low energies (Supplementary Figure 4a-e). Nevertheless, the rotation of the scattering pattern and twisted dispersion of the spin excitations can be clearly seen even in the raw data (Supplementary Figure 4a,d). The |**Q**|-dependent background was subtracted for the data in Fig. 2a-e following the practice of ref. 12. Magnetic signals symmetrically surrounding (0.5, 0.5) can be seen more clearly in the background-subtracted image (Fig. 2a-e). Raw data were presented at high energies when phonon excitations are weak (Fig. 2f-j).

Supplementary Note 5: Additional low energy data

Previous studies have suggested the presence of a weak magnetic order (ferromagnetic or antiferromagnetic) in $Li_{0.8}Fe_{0.2}OHFeSe$, which is likely related to the $Li_{0.8}Fe_{0.2}OH$ layer[10,13,14]. However, our data reveal no clear evidence of dispersive spin wave excitations associated with a static magnetic order. Supplementary Figure 5 illustrates the low energy excitation spectrum at 5 K in $Li_{0.8}Fe_{0.2}ODFeSe$. In addition to the incommensurate spin excitations near (0.5, 0.5±δ), indication of very weak excitations near (0.5, 0) was also observed at low energies (marked by arrows in Supplementary Figure 5a,b), which was no longer discernible at 45 meV (Supplementary Figure 5c). It is currently unclear whether these low energy excitations are related to the $Li_{0.8}Fe_{0.2}OD$ layer or FeSe layer, or they are non-magnetic. Further measurements are required to elucidate the nature of these excitations.

Supplementary References

[1] Kotliar, G. *et al.* Electronic structure calculations with dynamical mean-field theory. *Rev. Mod. Phys.* **78**, 865-951 (2006).

[2] Haule, K., Yee, C.-H. & Kim, K. Dynamical mean-field theory within the full-potential methods: electronic structure of $CeIrIn_5$, $CeCoIn_5$, and $CeRhIn_5$. *Phys. Rev. B* **81**, 195107 (2010).

[3] Blaha, P., Schwarz, K., Madsen, G. K. H., Kvasnicka, D. & Luitz, J. WIEN2k, an augmented plane wave + local orbitals program for calculating crystal properties, Techn. Universität Wien, Austria (2001).
[4] Perdew, J. P., Burke, K. & Ernzerhof, M. Generalized Gradient Approximation Made Simple. *Phys. Rev. Lett.* **77**, 3865-3868 (1996).
[5] Haule, K. Quantum Monte Carlo impurity solver for cluster dynamical mean-field theory and electronic structure calculations with adjustable cluster base. *Phys. Rev. B* **75**, 155113 (2007).
[6] Werner, P., Comanac, A., de Medici, L., Troyer, M. & Millis, A. J. Continuous-Time Solver for Quantum Impurity Models. *Phys. Rev. Lett.* **97**, 076405 (2006).
[7] Yin, Z. P., Haule, K., & Kotliar, G. Kinetic frustration and the nature of the magnetic and paramagnetic states in iron pnictides and iron chalcogenides. *Nat. Mater.* **10**, 932-935 (2011).
[8] Yin, Z. P., Haule, K., & Kotliar, G. Spin dynamics and orbital-antiphase pairing symmetry in iron-based superconductors. *Nat. Phys.* **10**, 845-850 (2014).
[9] Dong, X. L. *et al.* $(Li_{0.84}Fe_{0.16})OHFe_{0.98}Se$ superconductor: Ion-exchange synthesis of large single-crystal and highly two-dimensional electron properties. *Phys. Rev. B*, **92**, 064515 (2015).
[10] Lu, X. F. *et al.* Coexistence of superconductivity and antiferromagnetism in $(Li_{0.8}Fe_{0.2})OHFeSe$. *Nat. Mater.* **14**, 325-329 (2015).
[11] R. Coldea, computer code MSLICE, a data analysis program for time-of-flight technique neutron spectrometers (2004).
[12] Wang, Q. *et al.* Magnetic ground state of FeSe. *Nat. Commun.* **7**, 12182 (2016).
[13] Pachmayr, U. *et al.*, Coexistence of 3d-Ferromagnetism and superconductivity in $[(Li_{1-x}Fe_x)OH](Fe_{1-y}Li_y)Se$. *Angew. Chem., Int. Ed.* **54**, 293-297 (2015).
[14] Lynn, J. W. *et al.* Neutron investigation of the magnetic scattering in an iron-based ferromagnetic superconductor. *Phys. Rev. B* **92**, 060510(R) (2015).
